# Distancing Measures and Challenges Discussed by COVID-19 Outbreak Teams of Dutch Nursing Homes: The COVID-19 MINUTES Study

**DOI:** 10.3390/ijerph19116570

**Published:** 2022-05-27

**Authors:** Lisa S. van Tol, Hanneke J. A. Smaling, Wendy Meester, Sarah I. M. Janus, Sytse U. Zuidema, Margot W. M. de Waal, Monique A. A. Caljouw, Wilco P. Achterberg

**Affiliations:** 1 University Network for the Care Sector Zuid-Holland, Leiden University Medical Center, 2333 ZA Leiden, The Netherlands; h.j.a.smaling@lumc.nl (H.J.A.S.); ik.wendy@hotmail.com (W.M.); m.w.m.de_waal@lumc.nl (M.W.M.d.W.); m.a.a.caljouw@lumc.nl (M.A.A.C.); w.p.achterberg@lumc.nl (W.P.A.); 2 Department of Public Health and Primary Care, Leiden University Medical Center, 2333 ZA Leiden, The Netherlands; 3 Department of General Practice & Elderly Care Medicine, University of Groningen, University Medical Center Groningen, 9700 AD Groningen, The Netherlands; s.i.m.janus@umcg.nl (S.I.M.J.); s.u.zuidema@umcg.nl (S.U.Z.)

**Keywords:** COVID-19, nursing homes, infection prevention and control, isolation, distancing, qualitative

## Abstract

The most severe COVID-19 infections and highest mortality rates are seen among long-term care residents. To reduce the risk of infection, physical distancing is important. This study investigates what physical distancing measures were discussed by COVID-19 outbreak teams of Dutch long-term care organizations and what challenges they encountered. The COVID-19 MINUTES study is a qualitative multi-center study (*n* = 41) that collected minutes of COVID-19 outbreak teams from March 2020 to October 2021. Textual units about distancing measures were selected and analyzed using manifest content analysis for the first wave: early March–early May 2020; the intermediate period of 2020: mid-May–mid-September 2020; and the second wave: late September 2020–mid-June 2021. During all periods, COVID-19 outbreak teams often discussed distancing visitors from residents. Moreover, during the first wave they often discussed isolation measures, during the intermediate period they often discussed distancing staff and volunteers from residents, and during both the intermediate period and the second wave they often discussed distancing among residents. During all periods, less often admission measures were discussed. Challenges persisted and included unrest among and conflicts between visitors and staff, visitors violating measures, resident non-adherence to measures, and staffing issues. The discussed distancing measures and corresponding challenges may guide local long-term care and (inter)national policymakers during the further course of the COVID-19 pandemic, outbreaks of other infectious diseases, and long-term care innovations.

## 1. Introduction

The COVID-19 pandemic has caused millions of deaths worldwide, but the most severe COVID-19 infections and highest mortality rates are found among long-term care long-term care residents [1,2]. To reduce the risk of infections physical distancing measures that limit close interpersonal contact are important [3,4]. However, long-term care facilities such as nursing homes may require very specific physical distancing measures, for several reasons. First, age over 65 [5,6], underlying medical conditions, and increased vulnerability all are both common in long-term care and are linked to COVID-19 morbidity and mortality [1]. Second, large proportions of COVID-19-infected long-term care residents show atypical symptoms [7,8] or remain asymptomatic [9,10]. This complicates diagnosis and thus transmission prevention [9,10]. Third, a large proportion of long-term care residents has cognitive impairments such as dementia, which reduces their ability to understand and comply with distancing measures [11,12].

Consequently, much international policy recommendations and guidance documents about COVID-19 infection prevention and control (IPC) in long-term care facilities were formulated, e.g., by the World Health Organization [1], European Centre for Disease Prevention and Control [13], and the Centers of Disease Control and Prevention [14]. This guidance comprises, but is not limited to, the following distancing measures: isolation of residents with symptoms or confirmed COVID-19 [1,13,14], quarantining residents who had contact with confirmed cases [1], quarantining [14] or testing [1] residents upon (re)admission to the facility, maintaining physical distance of at least 1.5 m (six feet) from others [1,13,14], staff compartmentalization and cohorting [1], and strict visitor policies [1,13,14].

Besides policy recommendations and guidance documents, case studies have described responses to COVID-19 outbreaks in long-term care [15,16]. To date, large scale and longitudinal studies on physical distancing in long-term care during the COVID-19 pandemic are scarce [17]. The aims of this study are to investigate what physical distancing measures were discussed by COVID-19 outbreak teams of a large number of Dutch nursing homes during the first phases of the pandemic, and to investigate what corresponding challenges they encountered.

## 2. Materials and Methods

### 2.1. Design

The present study is part of the “COVID-19 management in nursing homes by outbreak teams” (MINUTES) study. In this multi-center study, minutes and other meeting documents of the central COVID-19 outbreak teams of Dutch long-term care organizations were collected from March 2020 through October 2021 [18]. A detailed description and first results of this study are published elsewhere [18].

### 2.2. Setting

Dutch long-term care organizations provide home care, and 24 h care in residential facilities including nursing homes for high-level or complex care and homes for assisted living care [19]. In this study, we focus on residential long-term care and refer to both types of residential facilities as nursing homes. In Dutch nursing homes, care is provided by multidisciplinary teams and coordinated by physicians specially trained in elderly care [20,21]. Dutch long-term care organizations usually have separate nursing home departments for residents with psychogeriatric disorders, such as dementia, and departments for residents that mainly suffer from somatic disorders [21,22]. With severe infectious disease outbreaks, Dutch long-term care organizations convert their infection prevention and control committees into, or install, central outbreak teams [18,23]. The central COVID-19 outbreak teams were multidisciplinary and often include management representatives, medical staff, policy advisors, support services, and communication specialists [18,23]. Some long-term care organizations also installed local COVID-19 outbreak teams per facility [18].

A national ‘first wave’ of coronavirus infections in the Netherlands was defined based on excess mortality from week 11 (early March) to week 19 (early May) of 2020 [24], and the indistinguishable second and third waves, hereafter referred to as ‘second wave’, based on excess mortality from week 39 (late September) 2020 to week 24 (mid-June) 2021 [25]. From 19 March to mid-May 2020, nursing homes were locked down for visitors [26]. From mid-May 2020, nursing homes were reopened and visitor policies were gradually eased [26]. From October 2020 the ‘COVID-19 (Temporary Measures) Act’ required nursing homes to remain open to visitors [27,28].

### 2.3. Sample and Data Collection

**Figure 1:**
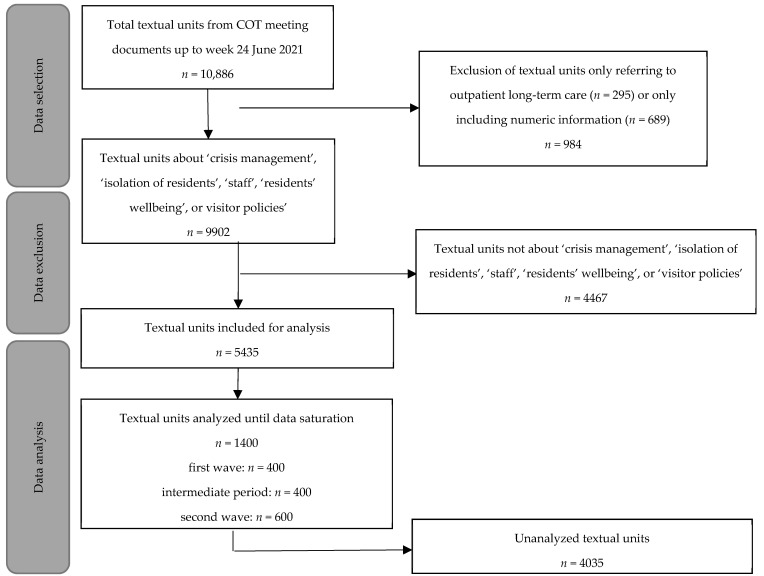
Flowchart of data selection, exclusion, and analysis.

The 41 participating Dutch long-term care organizations and their 41 central COVID-19 outbreak teams represented 563 nursing homes and almost 43,000 residents, which is over one third of nursing home residents nationwide [18]. These outbreak teams’ meeting documents were coded with a coding frame using a Castor online database [18,29]. With this primary coding frame, we included textual units coded in the topics crisis management, isolation, staff, residents’ wellbeing, and visitor policies until week 24 2021 (Figure 1). Textual units were pieces of text of one to a few sentences that relate to one measure, challenge, or other point of discussion [30].

### 2.4. Data Analysis

The included textual units were divided into ‘first wave’: week 11 (early March) to week 19 (early May) 2020; ‘intermediate period’: week 20 (mid-May) to week 38 (mid-September) 2020; and ‘second wave’: week 39 (late September) 2020 to week 24 (mid-June) 2021 to enable analysis of differences between these periods. Two researchers (LST and WM) independently coded the first 200 textual units of each time period. This was done inductively, without making use of the a priori identified topics. As coordinating researchers in the COVID-19 MINUTES study, LST and WM were familiar with the data. In several consensus meetings they discussed the coding of these in total 600 textual units, resolved discrepancies, and constructed a coding frame. Multiple codes could be assigned to a textual unit. The constructed coding frame was discussed with senior researchers HJAS and MAAC.

Subsequently, additional batches of 200 randomly selected textual units per time period were coded (LST). Repeatedly, previous batches were recoded when the coding frame was revised, and codes were clustered into categories of distancing measures [31]. New codes, and changes in the coding frame, were made by LST in consensus with HJAS. When no new codes emerged from a batch of textual units, we assumed that data saturation was reached for that time period [32,33]. Next, LST added subcodes to textual units coded as ‘challenge’. These subcodes were checked by HJAS followed by a consensus meeting (HJAS and LST). To confirm data saturation of challenges and (sub)categories of distancing, we checked if we covered the attention points in our weekly to triweekly summary reports [18].

Data was coded in Microsoft Excel software and exported to SPSS software (version 25 IBM corp., Armonk, New York, U.S.) for further analysis. Manifest content analysis was performed by using descriptive statistics to select textual units based on assigned time period and codes and to describe these. As is meant with manifest content analysis [30,31], throughout the coding and analyzing process we stayed close to terminology used in the data [34].

## 3. Results

Of the textual units from COVID-19 outbreak teams’ meeting documents collected until the end of the second wave (*n* = 10,886), 49.9% (*n* = 5435) were included. Of these, 1400 textual units were analyzed until data saturation (Figure 1). Distancing measures for visitors, staff and volunteers, and for residents among each other; admission measures; and isolation measures were identified (Table 1). Challenges with these measures (Table 2) were described in 83 of the 1400 textual units (5.9%). The results are limited to measures and challenges discussed by at least two outbreak teams in the analyzed data.

### 3.1. Distancing Visitors from Residents

Distancing measures for visitors were the most often discussed category of distancing. During all three periods, COVID-19 outbreak teams discussed visitor bans a few times: they maintained visitor bans (first wave), or reintroduced visitor bans in case of COVID-19 outbreaks (intermediate period) or when there was unrest about infections among managers or staff (second wave). Facilitating alternatives for visiting residents was often discussed during the first wave and hardly discussed during the second wave. These alternatives included visitor cabins or tents where residents could meet their relatives by appointment, window visits supported by a speaking–listening connection and aerial platforms, and video calling. A few times it was discussed that it was challenging to facilitate these alternatives.

Quote 1. “Family members of nursing home residents need more visual contact with the residents. The problem is: what requirements should such a meeting place comply with, and what can the nursing homes facilitate.”

(organization XB, first wave)

Restrictions to visiting were often discussed during the intermediate period and the second wave. First, visits often had to take place in residents’ rooms or outside (intermediate period). A few times reopening nursing homes’ restaurants for visitors was discussed (second wave). Second, a few times visiting was restricted to fixed daily visitor moments, sometimes spread out over the day. Third, numbers of visitors were limited to 1 or 2 per day during the first wave, or to 1–3 per day or per week during the intermediate period and the second wave. Exceptions were made for residents in the palliative or terminal phase of life. Fourth, several times the COVID-19 outbreak teams discussed instructing visitors about their measures including hand hygiene; escorting visitors directly to the resident; and asking visitors health questions or taking their temperature upon nursing home entry. Fifth, during the intermediate period several times the outbreak teams debated how to register visitors, e.g., via their website, an app, a list on paper at the nursing home’s entrance, or at the reception (intermediate period). Sometimes continuing registration of visitors was discussed and a few times lifting this measure was discussed (second wave).

Quote 2. “Closing restaurants on location is not a desirable option, because this would mean that all visits must take place in the (small) rooms. And that would mean extra activity and traffic on wards.”

(organization XH, second wave)

A challenge regarding distancing visitors from residents that was discussed during all three periods was dealing with residents’ relatives that violated measures, e.g., visitors taking their face masks off, ignoring instructions from staff, or not complying with the 1.5 m distance policy. Organizations reminded these visitors of their measures, deployed security guards, or denied entry to visitors who had violated measures. There was also unrest among visitors during all periods, mainly about full visitor bans. This unrest led to conflicts between staff and visitors. Furthermore, a few times outbreak teams discussed challenges related to implementing visitor restrictions in accordance with national and regional policies.

Quote 3. “organization sends a letter to the first contact persons of residents with the message that staff has the authority to withdraw visiting rights if family does not comply with the visiting rules.”

(organization XN, second wave)

### 3.2. Distancing Staff and Volunteers from Residents

To distance staff and volunteers from residents, COVID-19 outbreak teams often discussed building entry measures during the intermediate period and several times during the first and the second wave. During the intermediate period, entry for hairdressers was often discussed: some outbreak teams did “not yet” allow hairdressers, and some outbreak teams did allow hairdressers to enter the nursing home, a few times on the condition that they would see no other customers than their nursing homes’ residents. Moreover, during all periods a few times outbreak teams allowed volunteers access on a case-by-case basis. During all periods, it was discussed a few times that building entry was allowed for (para)medical care providers, such as dentists and pedicures, only in the case of emergency, when “medically necessary”, or on doctor’s orders.

Quote 4. “As long as the [nursing home] locations are closed, the hairdressers and pedicures will not start work. ADL [activities of daily living] care (for example dental hygienists) is allowed for prevention and on medical grounds.”

(organization YG, intermediate period)

In addition, several times during all three periods it was discussed that staff were compartmented to work in only one organization, location, or ward. Exceptions were made for practitioners who had to provide essential medical care in multiple locations. A few times COVID-19 outbreak teams discussed that they allowed staff to work in multiple wards if this was not on the same day, or to work in infected wards after they had worked in wards free from infection.

Quote 5. “Do not deploy staff against the compartmentation. If absolutely necessary: From clean to contaminated [ward]. Or after a 48 h interval.”

(organization XC, second wave)

### 3.3. Distancing among Residents

To distance residents from each other, group sizes were limited, residents from different living groups or departments were separated from each other, and 1.5 m distance was maintained between residents. These measures were mostly applied to activities including church services, eating in communal dining areas or restaurants, and visiting the garden and sitting at the terrace. During all three periods a few times COVID-19 outbreak teams increased (the severity of) distancing measures in response to local infection rates. During the intermediate period and the second wave, outbreak teams discussed some times that they allowed residents to meet each other again. A few times they indicated that it was challenging for residents to adhere to distancing from other residents.

Quote 6. “In view of the increase in the number of infections, allowing residents to participate in activities in other living rooms, or organizing joint activities with different wards is not a preferred option.”

(organization XZ, second wave)

Furthermore, COVID-19 outbreak teams discussed building entry measures for residents. During the first wave, a few times they discussed that if residents left the nursing home building, they would not be allowed to reenter the building. During the intermediate period, several times residents were allowed to leave the building to visit the hospital if this was medically necessary, or to go for a walk. A few times this was allowed on the condition or advice to maintain 1.5 m distance from others or else wear a face mask, avoid crowded places, and apply hand hygiene upon return. During the second wave, a few times outbreak teams discussed that they allowed residents to visit family and a few times they discussed that they advised against this.

### 3.4. Admission Measures

Temporary stops to admissions in nursing home locations with coronavirus infections were discussed a few times during all periods. Moreover, COVID-19 outbreak teams discussed restrictions to admitting new residents several times: it was discussed a few times that only one or two persons were allowed to accompany or help residents moving into the nursing home. New residents were tested for COVID-19, and were asked about or monitored for COVID-19 symptoms up to two weeks after admission. Furthermore, it was discussed that new residents had to stay in quarantine for 14 days (first wave), or for 1–10 days or until a negative COVID-19 test result was obtained (second wave).

A challenge discussed was scarce admission capacity for residents with psychogeriatric disorders. A few times outbreak teams discussed they did not admit these residents to locations that were not equipped for this resident group, or during outbreaks. Moreover, because adherence to quarantine was challenging for psychogeriatric residents, a few times outbreak teams requested home quarantine before admission, but discussed that this was not a feasible alternative as effective monitoring was not possible. Other challenges discussed a few times were hesitance of new residents about admission and increased numbers of crisis admissions. This hesitance was discussed to lead to empty beds, and to be due to visit restrictions and lack of a tour of the facility before admission.

Quote 7. “Psychogeriatric residents with strong urge to walk are difficult to keep in quarantine for 7 days, are therefore not admitted to [location], which is still ‘clean’.”

(organization XZ, first wave)

### 3.5. Isolation Measures

COVID-19 outbreak teams discussed isolation on COVID-19 units, cohort isolation, isolation in single rooms, and quarantine. Various terms were used for these different types of isolation and seemed to get mixed up at times (Table 1). During the first and the second wave, COVID-19 units were the most frequently discussed type of isolation, but during all three periods there was variation in when to transfer residents to COVID-19 units: a few times outbreak teams discussed transferring infected residents if they were the only infected resident within their ward, when other types of isolation were precluded, depending on the situation, or always unless otherwise decided. In addition, during the first wave a few times it was discussed when residents could be discharged from COVID-19 units back to regular wards: residents had to be free of symptoms for 24 h up to one week.

Quote 8. “[COVID-19 outbreak team] decides that if an infection is detected in a resident/client, he/she will be transferred to a cohort unless… Unless is always determined in consultation with the physician and [manager COVID-19 outbreak team].”

(organization XR, second wave)

Challenges for COVID-19 units were, first, meeting the demand for beds in COVID-19 units with fluctuating infection rates. This required insight into and reorganization of empty beds. Organizations worked together regionally to scale up and down the number of beds. Second, it was discussed a few times that the creation of COVID-19 units was not feasible in all nursing home locations, for example, due to the building structure.

Quote 9. “It is indicated that not all locations are suitable for [creating] a separate [COVID-19 unit]. The director of healthcare indicates that [person] should seriously think about this.”

(organization XX, second wave)

COVID-19 outbreak teams discussed that cohort isolation was applied to nursing home locations or wards when multiple residents were infected (all three periods), and for residents with the urge to walk (first wave). With both cohort isolation and COVID-19 units, staffing issues and impact on staff’s wellbeing were discussed: with COVID-19 units, a few times outbreak teams encountered staff shortages and employees unwilling to work with infected residents. Outbreak teams discussed several times that they restricted cohort entries to a limited number of staff members.

Quote 10. “cohort puts a lot of pressure on the staff roster. Cohort period is long. When cohort is dissolved, tension is expected when pressure is released. Aftercare is important.”

(organization XV, first wave)

Isolation in single rooms was the least often discussed type of isolation. COVID-19 outbreak teams called limiting the freedom of residents with isolation in a single room an “ethical” dilemma. Quarantine was the most often discussed type of isolation during the intermediate period. Besides quarantine upon admission to a nursing home (see admission measures), outbreak teams discussed a few times applying quarantine to residents upon readmission after they had left the nursing home building for a hospital visit or vacation (intermediate period), and several times to residents after they had contact with an infected person (intermediate period and the second wave). To lift quarantine, it was discussed several times during all periods that one or two COVID-19 tests needed to have a negative result.

A challenge with wandering residents, residents with severe behavioral problems or dementia, or “non-instructible” residents was adherence to isolation in single rooms and quarantine. A few times outbreak teams discussed how they customized quarantine measures or considered sedative medication to maintain isolation in single rooms. Moreover, a few times they considered establishing or had established separate sections in COVID-19 units for psychogeriatric residents (first and second wave).

Quote 11. “In case of a resident with the urge to walk and suspected to have COVID-19, the physician decides on how to best isolate the resident. In practice, this will involve confinement to the room or sedation.”

(organization XZ, first wave)

## 4. Discussion

This study investigated what physical distancing measures were discussed by COVID-19 outbreak teams of Dutch nursing homes during the first three periods of the COVID-19 pandemic and what corresponding challenges were encountered. Distancing visitors from residents was the most often discussed category of distancing during all periods. Moreover, these outbreak teams discussed isolation measures (often discussed during the first wave), distancing staff and volunteers from residents (often discussed during the intermediate period), distancing among residents (often discussed during the intermediate period and second wave), and admission measures (less often discussed). The distancing measures for visitors, staff and volunteers, and for residents among each other all included nursing home entry measures and measures for physical distancing within nursing homes or wards. For both distancing measures for staff and volunteers and for residents among each other, very few challenges were encountered.

COVID-19 circumstances in (inter)national long-term care settings varied, making comparison complex. Still, we observed mainly similarities between our findings and cross-national policies and recommendations [1,13,14,35,36]. When comparing our results with the scientific literature, there are some similarities and differences. First, in line with our findings, a literature review showed that visitor policies and physical distancing among residents were among the most common COVID-19 guidelines for long-term care [37]. Visitor bans and the shift to allowing restricted visiting were advised or made compulsory by various governments and non-governmental agencies [28,38]. Organizing restricted visiting was more frequently discussed by COVID-19 outbreak teams than maintaining full bans, and, according to another Dutch study, increased workload for staff [39]. In contrast to our results, the latter study also reported compliance and no major incidents with local visiting policies in Dutch nursing homes [39]. In the minutes, many challenges with visitors were described. Long-term care organizations sometimes acted hard against visitors violating visiting restrictions. To prevent these challenges with visitors and the increase in staff workload in the future, local and (inter)national policy makers may carefully consider staffs’, visitors’, and residents’ experiences with and perspectives on visiting policies. This may lead to a better balance between impact on their daily lives and infection prevention.

Second, isolation measures were recommended in many countries [40] and the four types of isolation we identified have been implemented internationally [17]. During the first wave, COVID-19 outbreak teams often discussed isolation measures, but during the second wave other distancing measures were more often discussed. However, the challenges encountered with isolation measures during the first wave were also discussed during the second wave. This suggests that the challenges with isolation measures were hard to solve in the short term. For future outbreaks of COVID-19 and other infectious diseases, we suggest that policy makers in long-term care innovation should involve nursing homes staff and pay attention to the feasibility of creating COVID-19 units, fluctuating needs for beds, staffing cohorts or COVID-19 units, impact on staff wellbeing and residents’ freedom. In the short term, local policy makers may only implement isolation measures that suit their building structure, staff, and resident group; consider regional collaboration regarding isolation; and also focus on other types of distancing. In addition, non-adherence to isolation measures, customization of quarantine measures, and implementation of cohort isolation mainly concerned residents with psychogeriatric disorders. This indicates that policy makers may better tailor isolation measures to this resident group. The challenges that include limiting freedom [41], staffing [41], and affecting staff wellbeing [26,41] have been mentioned elsewhere, although not earlier supported by primary data.

Third, measures concerning admission of new residents continued to be discussed, although less often than other measures, and were recommended in half of 30 Western countries [40].

Distancing measures are implemented next to each other, and next to hygiene measures, use of personal protective equipment (PPE), symptom monitoring, testing, and vaccination [16,18,42]. In this study only textual units from COVID-19 outbreak teams’ meeting documents describing distancing measures were included. However, according to literature, on the one hand, the focus on distancing measures increased with insufficient testing possibilities and PPE availability [9,15]. On the other hand, distancing measures can be targeted to infected residents who can be identified with testing and symptom monitoring [15,43,44]. Future studies should explore the balance between various IPC measures.

A limitation of this study is that the indirect observational nature limits our insights into possible bias regarding what parts of COVID-19 outbreak team meetings were documented. The minutes were sometimes brief descriptions of decisions that seemed to lack context [18]. This may also explain the relatively low numbers of textual units describing challenges. Furthermore, content analysis enabled us to count textual units. These counts provide insight into the focus of COVID-19 outbreak teams on distancing measures in relation to each other during the first periods of the COVID-19 pandemic. However, they reflect only analyzed batches of data from time periods of different lengths, and do not necessarily reflect implementation frequency or importance of distancing measures [31]. Developments in national guidelines will have influenced the outbreak teams’ focus.

Strengths of this study stem from the novel method of qualitative data collection. First, collecting existing meeting documents enabled data collection from the large sample of 41 participating COVID-19 outbreak teams, representing over 500 nursing homes [18]. Second, the longitudinal nature of data collection led to a complete overview of distancing measures that were discussed, also in between waves of infections. Most other studies only described what measures were applied during COVID-19 outbreaks [17]. These studies may have missed distancing measures that were applied preventively, prior to outbreaks. Third, performing content analysis on these meeting documents enabled generation of big qualitative data without adding to the workload of nursing home staff during the turbulent first periods of the COVID-19 pandemic. Summary reports served as quick input for local and national policy during the study period [18], while results of more in-depth analysis may also offer guidance beyond the COVID-19 pandemic.

## 5. Conclusions

This study investigated what distancing measures and corresponding challenges were discussed by COVID-19 outbreak teams in Dutch nursing homes. Measures to distance visitors from residents, distance staff and volunteers from residents, distance residents among each other, admission measures, and isolation measures were discussed. Challenges with distancing measures persisted over time and included, but were not limited to, unrest among and conflicts between visitors and staff, visitors violating measures, resident non-adherence to measures, and staffing issues.

Since most distancing measures described were in accordance with (inter)national policies and policy recommendations, these apparently offered helpful guidance. However, the shifts in COVID-19 outbreak teams’ discussions over time and persisting discussion about challenges may indicate that long-term care organizations also continuously learned lessons from experience. To provide more context to our findings and further reveal what lessons were learned, our findings should be discussed with stakeholders. The distancing measures and challenges discussed may guide and inspire long-term care organizations and (inter)national and local policymakers during the further course of the COVID-19 pandemic, future infectious disease outbreaks, and in long-term care innovations.

## Figures and Tables

**Table 1:** Textual units per category of distancing measures discussed by COVID-19 outbreak teams.

Categories of Distancing Measures, Subcategories of Distancing Measures *	First Wave *n* = 400	Intermediate Period *n* = 400	Second Wave *n* = 600
**Distancing visitors from residents**	57 (14.25%)	113 (28.25%)	105 (17.5%)
Ban on visitors	5	4	3
Facilitation of alternatives to visiting	20	13	1
Assigned place for visiting	2	12	12
Fixed visiting times	1	10	4
Health checks for visitors	3	8	1
Limited numbers of visitors allowed	9	18	32
Receiving and instructing visitors	3	9	4
Registration of visitors	1	15	14
**Distancing staff and volunteers from residents**	29 (7.25%)	44 (11.00%)	27 (4.50%)
Building entry measures	15	33	8
Compartmenting staff	13	11	11
**Distancing among residents**	21 (5.25%)	49 (12.25%)	58 (9.67%)
Building entry measures	14	24	13
Limited group size	2	3	15
Keeping (living-)groups separate	1	5	13
1.5 m distance	2	5	11
**Admission measures**	33 (8.25%)	24 (6.00%)	40 (6.67%)
Admission stops	4	5	3
Health checks	2	1	2
Quarantine	9	4	12
Moving with a limited number of people	5	3	1
Testing	4	1	8
**Isolation measures**	54 (13.50%)	24 (6.00%)	41 (6.83%)
Isolation on COVID-19 units †	30	11	25
Cohort isolation ‡	17	8	19
Isolation in single rooms	7	3	6
Quarantine (in other situations then at admission)	4	16	20

* textual units could be coded with single or multiple codes for (sub)categories of distancing; † other terms used were Corona-unit, COVID ward, (corona-)cohort, cohort ward, and cohort- or corona location; ‡ other terms used were cohort nursing, cohort ward, and cohorting.

**Table 2:** Textual units regarding challenges discussed by COVID-19 outbreak teams per category of distancing measures.

Challenges per Category of Distancing Measures	First Wave *n* = 400	Intermediate Period *n* = 400	Second Wave *n* = 600
**Distancing visitors from residents**			
Compliance to national policies	1	N/A	3
Facilitating alternatives to visits	1	2	N/A
Unrest among and conflicts between visitors and staff	2	2	3
Visitors violating measures	2	2	6
**Distancing staff and volunteers from residents**	N/A	N/A	N/A
**Distancing among residents**			
Resident non-adherence	1	N/A	1
**Admission measures**			
Increased number of crisis admissions	1	1	N/A
Limited admission capacity for psychogeriatric residents	2	N/A	3
New residents’ hesitance about admission and empty beds	N/A	1	2
Unfeasibility of quarantine at home	N/A	1	1
**Isolation measures**			
Non-feasibility to create COVID-19 units	N/A	1	1
Fluctuating need for beds	N/A	N/A	2
Impact on staff’s wellbeing	2	N/A	1
Staffing issues	3	N/A	3
Ethical dilemma of limiting residents’ freedom	1	N/A	1
Resident non-adherence	3	2	2

## Data Availability

The data presented in this study are available on request from the corresponding author. The data are not publicly available due to the agreement with participating organizations. During the consent process, participating organizations were explicitly guaranteed that the data would be pseudonymized by the study’s research center and that pseudonymized data would only be seen by members of the study team. For any discussions about the dataset, please contact UNC-ZH@lumc.nl.
